# Adverse event signal mining and serious adverse event influencing factor analysis of fulvestrant based on FAERS database

**DOI:** 10.1038/s41598-024-62238-1

**Published:** 2024-05-18

**Authors:** Guisen Yin, Guiling Song, Shuyi Xue, Fen Liu

**Affiliations:** 1 Department of Pharmacy, The Affiliated Cancer Hospital of Xiangya School of Medicine, Central South University/Hunan Cancer Hospital, Changsha, 410011 Hunan China; 2 Department of Pharmacy, Yantai Hospital of Traditional Chinese Medicine, YantaiShandong, 264000 China; 3 Department of Chemical Medicine, Yantai Center for Food and Drug Control, YantaiShandong, 264003 China; 4 Department of Pharmacy, Qingdao Central Hospital, University of Health and Rehabilitation Sciences (Qingdao Central Medical Group), Qingdao, 266042 Shandong China

**Keywords:** Fulvestrant, Pharmacovigilance, Adverse events, Real-world, Data mining, FAERS, Cancer, Breast cancer

## Abstract

Fulvestrant, as the first selective estrogen receptor degrader, is widely used in the endocrine treatment of breast cancer. However, in the real world, there is a lack of relevant reports on adverse reaction data mining for fulvestrant. To perform data mining on adverse events (AEs) associated with fulvestrant and explore the risk factors contributing to severe AEs, providing a reference for the rational use of fulvestrant in clinical practice. Retrieved adverse event report information associated with fulvestrant from the U.S. Food and Drug Administration (FDA) Adverse Event Reporting System (FAERS) database, covering the period from market introduction to September 30, 2023. Suspicious AEs were screened using the reporting odds ratio (ROR) and proportional reporting ratio methods based on disproportionality analysis. Univariate and multivariate logistic regression analyses were conducted on severe AEs to explore the risk factors associated with fulvestrant-induced severe AEs. A total of 6947 reports related to AEs associated with fulvestrant were obtained, including 5924 reports of severe AEs and 1023 reports of non-severe AEs. Using the disproportionality analysis method, a total of 210 valid AEs were identified for fulvestrant, with 45 AEs (21.43%) not listed in the product labeling, involving 11 systems and organs. The AEs associated with fulvestrant were sorted by frequency of occurrence, with neutropenia (325 cases) having the highest number of reports. By signal strength, injection site pruritus showed the strongest signal (ROR = 658.43). The results of the logistic regression analysis showed that concurrent use of medications with extremely high protein binding (≥ 98%) is an independent risk factor for severe AEs associated with fulvestrant. Age served as a protective factor for fulvestrant-related AEs. The co-administration of fulvestrant with CYP3A4 enzyme inhibitors did not show statistically significant correlation with the occurrence of severe AEs. Co-administration of drugs with extremely high protein binding (≥ 98%) may increase the risk of severe adverse reactions of fulvestrant. Meanwhile, age (60–74 years) may reduce the risk of severe AEs of fulvestrant. However, further clinical research is still needed to explore and verify whether there is interaction between fulvestrant and drugs with high protein binding through more clinical studies.

## Introduction

Breast cancer (BC) is the most common malignant tumor that poses a significant threat to women’s health and life. Its incidence and mortality rates have been increasing year by year in China. The most common subtype of breast cancer is estrogen receptor (ER) positive and human epidermal growth factor receptor 2 (HER2) negative. Long-term treatment for this subtype relies on endocrine therapy (ET)^1^. Endocrine therapy for breast cancer mainly includes aromatase inhibitors (AIs), selective estrogen receptor modulators (SERMs), and selective estrogen receptor degraders (SERDs)^2^. Fulvestrant, as the world’s first SERD, has gained wide attention. It was first approved by the FDA on April 25, 2002, for the treatment of postmenopausal women with hormone receptor-positive advanced breast cancer that had progressed after anti-estrogen therapy^3^. It entered the Chinese market on June 4, 2010^4^.

Since estrogen exerts its effects by binding to estrogen receptors, when fulvestrant competitively binds to estrogen receptors, estrogen has no chance to attach to breast cells and regulate their growth and reproduction, thereby achieving the goal of treating breast cancer. Fulvestrant can also change the shape of estrogen receptors by reducing their quantity in breast cells, rendering them unable to function properly^5,6^. Clinical studies have shown that in hormone receptor-positive, postmenopausal metastatic breast cancer patients, Fulvestrant has comparable or superior effects on slowing down or inhibiting cancer growth compared to aromatase inhibitors like anastrozole, when tamoxifen is no longer effective^7^.

Fulvestrant inevitably causes drug-related adverse reactions while treating breast cancer. A review of medical records of 171 patients with locally advanced or metastatic HR+/HER2- breast cancer who received fulvestrant treatment showed that 66.9% of patients experienced drug-related adverse reactions, with severe adverse reactions accounting for 8.6% of cases^8^. In recent years, there has been growing concern about adverse reactions in endocrine therapy for breast cancer. However, in the real world, there is a lack of relevant reports on adverse reaction data mining for fulvestrant. Therefore, our study aims to analyze the safety of fulvestrant by exploring the latest data from the FAERS database, in order to provide reference for rational clinical medication.

## Materials and methods

### Data source

The FAERS database is a publicly accessible, spontaneous reporting system created and maintained by the FDA. The database is primarily used to monitor the safety of drugs and biologics that are marketed in the United States^9^. It includes information on drug AEs and medication errors, to some extent reflecting the occurrence of real-world drug-related AEs. The FAERS database is widely used for drug safety evaluations and has become an important tool for pharmacovigilance analysis^10^. In this study, we downloaded all data from the first quarter of 2004 to the third quarter of 2023^11^. These data include seven sections: patient demographics and management information (DEMO), AEs (REAC), patient outcomes (OUTC), drug information (DRUG), drug therapy start and end dates (THER), report sources (RPSR), and use/diagnosis indications (INDI). The DRUG section contains primary suspected drugs, secondary suspected drugs, concomitant drugs, or interacting drugs.

### Preprocessing

Due to the fact that the FAERS database is a system of voluntary reports from healthcare professionals and consumers, it contains many duplicate reports and cases with missing information. Therefore, we need to further filter and remove these data. To identify and remove duplicate reports, we selected the PRIMARYID, CASEID, and FDA_DT fields from the DEMO table and sorted them. When the CASEID is the same, we only kept the report with the most recent date. When the CASEID and FDA_DT are the same, we kept the report with the largest PRIMARYID value. In addition, since the first quarter of 2019, each quarterly data package includes a list of deleted reports. After removing duplicate data, we deleted reports based on the CASEID listed in the list of deleted reports. In order to ensure the accuracy and validity of the data, we removed cases with missing values, such as gender, age, reporting country, reporter’s occupation, reporting outcomes, drug adverse reaction information, and reports with missing drug information. To reduce the impact of “indication bias” (i.e., reporting the indication for prescription drugs as ADR), this study excluded PTs (Preferred Term), related to indications for the use of fulvestrant from the analysis.

In the FAERS database, drug names are not standardized, including International Nonproprietary Names, Trade Names, and abbreviations. We standardized the drug names in the DRUG table using OHDSI. We filtered adverse event reports with fulvestrant as the primary suspected drug. When processing AEs in the “REAC” table, we followed the Medical Dictionary for Regulatory Activities (MedDRA Version 24.0). MedDRA includes five levels of results, namely System Organ Class (SOC), High-Level Group Term (HLGT), High-Level Term (HLT), PT (Preferred Term), and Lowest Level Term (LLT)^12^. We mapped the Adverse Event (AE) names in the REAC table using MedDRA 24.0.

We categorized adverse event outcomes of fulvestrant into two groups: severe AEs and non-severe AEs. The severe AEs group encompasses occurrences of death, life-threatening situations, hospitalization or prolonged hospital stay, permanent or severe disability or impairment, congenital anomalies, or occurrences of defective medical events. If none of the above AEs occurred, they were included in the non-serious AEs group. Additionally, when a single report contained multiple AEs, if any of the above serious AEs occurred, they were included in the serious AEs group.

### Data mining

We utilized the ROR method and the PRR method to evaluate the occurrence ratio of AEs for fulvestrant compared to all other drugs excluding fulvestrant. For the identification of a significant risk signal for fulvestrant, the following criteria must be met: (1) ROR method: the number of adverse event reports is ≥ 3, and the lower limit of the 95% confidence interval (CI) is > 1; (2) PRR method: the number of adverse event reports is ≥ 3, the PRR is > 2, and the χ^2^ ≥ 4^13^. These methods are based on the proportional imbalance in the 2 × 2 contingency table, as shown in Supplementary Table S1 and Supplementary Table S2 outlines the formulas for ROR and PRR and the conditions for signal generation. In general, the higher the values of ROR and PRR, the stronger the signal, indicating a stronger statistical association between the target drug and the target adverse event^14^.

### Statistical analysis

We performed statistical analysis using SPSS version 26.0 (SPSS Inc., Chicago, IL, USA). The frequency data were described using counts and proportions. We selected severe AEs and conducted both univariate and multivariate logistic regression analyses to determine the odds ratios (ORs) of fulvestrant-induced severe AEs under different exposure factors such as gender, age, and concomitant medications. *p* < 0.05 indicated a statistically significant difference.

## Results

### The reporting situation of fulvestrant-related AEs

Through the FAERS database, we retrieved a total of 6947 AEs related to fulvestrant (Table 1). From the distribution of report years, the number of reported cases showed an increasing trend over the years. In terms of gender composition, there were more female cases (6534) than male cases (99). After excluding 1476 cases with unknown age, the age group of 60–74 years accounted for the highest proportion (33.64%), followed by the 45–59 age group at 22.31%. Among the 6947 cases, physicians submitted 4942 reports of AEs, accounting for 71.14%. In terms of reporting countries, the top five countries with the highest number of reports were the United States, Germany, France, Canada, and Japan, accounting for 55.71% of the total reports. Hospitalization or prolonged hospital stay (n = 1782, 25.65%) was the most common outcome reported for AEs. In terms of indications, the majority of reports were for breast cancer patients (51.22%), followed by breast cancer metastasis at 29.44%. When considering comorbidities, the top five reported conditions were hypertension, diabetes, gastroesophageal reflux disease, hypothyroidism, and depression. Regarding concomitant medications, there were 3007 reports of fulvestrant used concomitantly with other drugs, with the highest reported concomitant drug being trastuzumab (5.12%), followed by aspirin (2.75%). The ranking of the top 10 concomitant medications is shown in Table 2.

**Table 1:** Clinical characteristics of patients treated with fulvestrant in the FAERS database.

Characteristic		Reports, N (%)
Age	< 18	22 (0.31)
	≥ 18, < 45	363 (5.23)
	≥ 45, < 60	1550 (22.31)
	≥ 60, < 75	2337 (33.64)
	≥ 75	1199 (17.26)
	Not specified	1476 (21.25)
Gender	Male	99 (1.43)
	Female	6534 (94.05)
	Not specified	314 (4.52)
Reporter	Consumer	1864 (26.83)
	Healthcare professional	4942 (71.14)
	Not specified	141 (2.03)
Reporting region (TOP 5)	Americas	2108 (30.34)
	Germany	767 (11.04)
	France	410 (5.90)
	Canada	298 (4.29)
	Japan	287 (4.13)
Seriousness	Non-serious	1023 (14.73)
	Serious	5924 (85.27)
Outcome	Died	1439 (20.71)
	Life threatening	66 (0.95)
	Disabled	155 (2.23)
	Hospitalized	1782 (25.65)
indication	Breast cancer	3558 (51.22)
	Breast cancer metastasis	2045 (29.44)
	Bone cancer	29 (0.42)
	Lung cancer	22 (0.32)
	Ovarian cancer	15 (0.22)
	Uterine cancer	9 (0.13)
	Liver cancer	7 (0.10)
	Not specified	126 (18.17)
Complication (TOP 5)	Hypertension	404 (5.82)
	Diabetes	159 (2.23)
	Gastroesophageal reflux disease	102 (1.47)
	Hypothyroidism	102 (1.47)
	Depression	101 (1.45)
Event year	2000–2005	120 (1.73)
	2006	54 (0.78)
	2007	71 (1.02)
	2008	79 (1.14)
	2009	81 (1.17)
	2010	67 (0.96)
	2011	111 (1.60)
	2012	178 (2.23)
	2013	155 (2.23)
	2014	153 (2.20)
	2015	264 (3.80)
	2016	335 (4.82)
	2017	525 (7.56)
	2018	573 (8.25)
	2019	692 (9.96)
	2020	928 (13.36)
	2021	979 (14.09)
	2022	927 (13.34)
	2023	655 (9.43)

**Table 2:** Top 10 concomitant drugs.

Concomitant medication	Reports, N (%)	Concomitant medication	Reports, N (%)
Denosumab	356 (5.12)	Atorvastatin	124 (1.78)
Aspirin	191 (2.75)	Paracetamol	120 (1.73)
Zoledronic acid	162 (2.33)	Acetaminophen	119 (1.71)
Vitamin D3	150 (2.16)	Exemestane	110 (1.58)
Omeprazole	125 (1.80)	Metformin	109 (1.57)

### Signal monitoring results for fulvestrant-related AEs

A total of 6947 reports of adverse reactions related to fulvestrant involved 1895 potential risk signals, and after calculation using PRR and ROR, a total of 210 valid risk signals were obtained. See Supplementary Table 3 for details. These valid risk signals were then ranked in descending order based on their occurrence frequency. The top 10 valid risk signals associated with fulvestrant, listed in descending order of frequency, were neutropenia, injection site itching, fatigue, cough, decreased appetite, thrombocytopenia, hair loss, back pain, leukopenia, and decreased platelet count (Table 3). Furthermore, the ROR intensity was used to rank the top 10 valid risk signals associated with fulvestrant. Arranged in descending order of ROR strength, the top 10 valid risk signals were injection site itching, prolonged QT interval on electrocardiogram, skin toxicity, abnormal liver enzymes, anxiety, bronchospasm, acute interstitial pneumonia, abnormal platelet count, decreased sensation at the injection site, and allergic-like reactions (Table 4).

**Table 3:** Top 10 adverse events by report number.

PT	Reports	ROR	PRR (χ^2^)
Neutropenia	325	10.30 (9.20–11.52)	9.80 (2559.28)
Injection site pain	205	2.65 (2.30–3.04)	2.59 (201.19)
Asthenia	186	2.09 (1.81–2.42)	2.06 (101.31)
Cough	183	2.88 (2.48–3.33)	2.82 (215.22)
Decreased appetite	146	2.62 (2.22–3.09)	2.58 (140.82)
Thrombocytopenia	126	4.89 (4.10–5.83)	4.82 (376.89)
Alopecia	119	2.33 (1.94–2.79)	2.30 (87.06)
Back pain	115	2.06 (1.71–2.47)	2.04 (60.01)
Leukopenia	114	10.06 (8.35–12.11)	9.89 (898.56)
Pulmonary embolism	82	3.01 (2.42–3.74)	2.98 (106.20)

**Table 4:** Top 10 adverse events with ROR values.

PT	Reports	ROR	PRR (χ^2^)
Injection site pruritus	24	658.43 (413.51–1048.40)	655.83 (11,161.44)
Electrocardiogram QT prolonged	31	529.28 (355.16–788.78)	526.58 (12,304.34)
Skin toxicity	14	367.47 (207.18–651.78)	366.63 (3973.03)
Hepatic enzyme abnormal	4	290.27 (101.27–831.97)	290.08 (763.75)
Anxiety	37	264.87 (187.60–373.96)	263.26 (8253.92)
Bronchospasm	14	191.72 (110.58–332.42)	191.28 (2236.24)
Acute interstitial pneumonitis	6	145.18 (63.26–333.20)	145.04 (668.77)
Platelet count abnormal	5	140.82 (56.74–349.51)	140.71 (521.90)
Injection site hypoaesthesia	7	125.84 (58.53–270.58)	125.7 (699.09)
Anaphylactoid reaction	6	115.55 (50.66–263.59)	115.44 (538.06)

Compared with the drug label, many AEs that were not mentioned in the drug label were identified in Table 5. A total of 45 new AEs were identified, involving 11 different systems and organs, including but not limited to bone marrow failure, small intestinal obstruction, acute respiratory failure, laryngeal edema, painful respiration, jaundice, hepatic necrosis, and hypertensive crisis.

**Table 5:** Adverse events not documented in the label of fluvastatin.

SOC	PT	Reports	ROR	PRR (χ^2^)
Blood and lymphatic system disorders	Pancytopenia	32	2.50 (1.76–3.54)	2.49 (27.08)
	Full blood count decreased	16	3.39 (2.07–5.54)	3.38 (24.49)
	Bone marrow failure	8	9.81 (4.89–19.65)	9.80 (54.41)
Cardiac disorders	Pericardial effusion	16	2.84 (1.74–4.65)	2.84 (17.26)
	Bundle branch block left	4	3.78 (1.42–10.08)	3.78 (5.61)
	Intracardiac thrombus	4	3.90 (1.46–10.40)	3.90 (5.95)
Gastrointestinal disorders	Ascites	59	3.90 (1.46–10.40)	8.44 (378.20)
	Frequent bowel movements	11	2.10 (1.16–3.80)	2.10 (5.28)
	Eructation	10	2.33 (1.25–4.33)	2.32 (6.27)
	Small intestinal obstruction	9	3.47 (1.80–6.68)	3.47 (4.49)
Respiratory, thoracic and mediastinal disorders	Interstitial lung disease	54	4.85 (3.71–6.34)	4.82 (159.40)
	Epistaxis	47	2.51 (1.88–3.34)	2.50 (40.78)
	Bronchospasm	14	4.23 (2.50–7.15)	4.22 (31.24)
	Acute respiratory failure	9	2.20 (1.14–4.23)	2.20 (4.72)
	Laryngospasm	9	13.69 (7.10–26.39)	13.67 (92.76)
	Acute interstitial pneumonitis	7	50.55 (22.46–113.78)	50.51 (237.37)
	Laryngeal oedema	5	3.25 (1.35–7.82)	3.25 (5.69)
	Painful respiration	3	5.14 (1.65–15.95)	5.13 (6.26)
Skin and subcutaneous tissue disorders	Palmar-plantar erythrodysaesthesia syndrome	16	2.49 (1.52–4.07)	2.49 (12.75)
	Skin odour abnormal	10	9.64 (5.18–17.95)	9.63 (68.56)
	Skin hypopigmentation	5	16.24 (6.73–39.18)	16.23 (56.53)
	Rash vesicular	4	3.68 (1.38–9.83)	3.68 (5.35)
	Skin indentation	4	39.51 (14.67–106.39)	39.49 (111.68)
	Trichorrhexis	3	4.24 (1.37–13.16)	4.24 (4.52)
Hepatobiliary disorders	Jaundice	26	3.59 (2.44–5.28)	3.58 (45.68)
	Hepatomegaly	13	5.16 (2.99–8.90)	5.15 (39.32)
	Bile duct stenosis	5	19.62 (8.12–47.36)	19.60 (69.90)
	Hepatic cyst	4	5.29 (1.98–14.11)	5.29 (9.91)
	Hepatic necrosis	4	3.83 (1.44–10.22)	3.83 (5.76)
Vascular disorders	Lymphoedema	18	11.71 (7.36–18.63)	11.68 (164.27)
	Hypertensive crisis	16	5.53 (3.38–9.03)	5.52 (54.58)
	Circulatory collapse	12	2.63 (1.49–4.64)	2.63 (10.53)
Metabolism and nutrition disorders	Hyperglycaemia	67	7.45 (5.85–9.48)	7.38 (362.00)
	Hypokalaemia	22	2.17 (1.43–3.30)	2.17 (12.67)
	Iron deficiency	7	8.26 (3.93–17.36)	8.25 (27.48)
	Polydipsia	6	5.54 (2.48–12.34)	5.53 (17.92)
Investigations	Ejection fraction decreased	10	2.38 (1.28–4.42)	2.38 (6.64)
	Glomerular filtration rate decreased	9	3.48 (1.81–6.70)	3.48 (13.47)
	Blood calcium increased	8	3.73 (1.86–7.46)	3.72 (13.30)
Infections and infestations	Erysipelas	10	9.53 (5.12–17.75)	9.52 (67.62)
	Injection site abscess	9	13.75 (7.13–26.50)	13.73 (93.20)
	Injection site cellulitis	5	10.45 (4.34–25.17)	10.44 (33.57)
	Pustule	4	10.45 (3.91–27.93)	10.45 (25.23)
Immune system disorders	Decreased immune responsiveness	9	4.31 (2.24–8.30)	4.31 (19.62)

### Factors influencing severe AEs associated with fulvestrant

Analysis of the correlation between patient gender, age, and concomitant medication with severe AEs associated with fulvestrant was presented in Table 6. Univariate and multivariate logistic regression analysis indicated that age and concomitant medication may be influencing factors for the occurrence of severe AEs with fulvestrant (*p* < 0.05). Our study found that the risk of severe AEs related to fulvestrant increased by 1.522 times when co-administered with drugs exhibiting extremely high protein binding (≥ 98%) [OR = 1.522 (1.173, 1.975), *p* = 0.002]. The risk of severe AEs related to fulvestrant for patients aged 60–74 was 0.521 times that of the 0–44 age group [OR = 0.521 (0.296, 0.916), *p* = 0.024]. The concomitant use of CYP3A4 enzyme inhibitors was not found to be an independent risk factor for severe AEs related to fulvestrant. Refer to Table 6 for details.

**Table 6:** Univariate and multivariate logistic regression analysis of the odds ratio for fulvestrant-related fatal adverse events.

Variable	Factor	Univariate analysis		Multivariate analysis	
		*p*	OR (95%CI)	*p*	OR (95%CI)
Gender	Male (reference)	–	1	–	–
	Female	0.647	0.49 (0.217, 2.582)	–	–
Age	0–44 (reference)	–	1	–	1
	45–59	0.562	0.840 (0.466, 1.515)	0.453	0.797 (0.441, 1.441
	60–74	**0.03**	**0.534 (0.308, 0.941)**	**0.024**	**0.521 (0.296, 0.916)**
	≥ 75	0.09	0.604 (0.338, 1.082)	0.073	0.586 (0.327.1.050)
Concomitant drug (protein binding)	No (reference)	–	1	–	1
	< 85%	0.23	1.212 (0.885, 1.659)	0.202	1.228 (0.896, 1.682)
	≥ 85, < 98	0.319	0.867 (0.656, 1.147)	0.296	0.861 (0.650, 1.140)
	≥ 98	**0.001**	**1.546 (1.193, 2.005)**	**0.002**	**1.522 (1.173, 1.975)**
Concomitant drug (CYP3A4 inhibitor)	No (reference)	/	1	–	–
	Others	0.075	1.213 (0.981, 1.500)	–	–
	CYP3A4 inhibitor	0.164	1.364 (0.881, 2.113)	–	–

Significant values are in bold.

## Discussion

### Analysis of fulvestrant AEs profile

The clinical characteristics of fulvestrant-associated AEs may be related to the population affected by breast cancer. According to the latest data, there will be approximately 2790 new cases of male breast cancer and 313,210 new cases of female breast cancer in the United States in 2023. The incidence rate of breast cancer in females was approximately 112 times higher than in males^13^. Our study found that there were significantly more AE reports from female patients compared to male patients (6534 and 99, respectively), which was consistent with the incidence of new cases of breast cancer. In terms of age, the incidence of breast cancer was higher in the 60–75 age group, followed by the 45–59 age group, with a median age of onset at 65 years. This was largely consistent with data from the American Cancer Society’s statistical center^15^. Our study found that approximately 93% of breast cancer patients are aged 45 and above, especially those in the 45–74 age group, accounting for 70% of the cases. This may be related to factors such as family history of breast cancer, radiation exposure, genetic mutations, among others. In female breast cancer patients, hormone changes around menopause, reproductive history, and frequent hormone use are the main contributory factors^16^. The data source in the FAERS was predominantly from the United States, reflecting the fulvestrant market situation in the country. The indications for fulvestrant are generally consistent across different countries, and there are no significant off-label uses reported. Over 80% of the reported indications were related to breast cancer or breast cancer metastasis, which is largely consistent with the product information for fulvestrant. The combination of denosumab or zoledronic acid with fulvestrant is frequently employed in patients with breast cancer bone metastases due to their bone-protective properties. Other drugs such as omeprazole, pantoprazole, metformin, atorvastatin, acetaminophen, and aspirin may be associated with the comorbidities of the patients, such as gastroesophageal reflux disease, hypertension, and diabetes. Since fulvestrant is an injectable formulation primarily used in healthcare facilities, the majority of AE reports come from healthcare professionals (71.14%), with fewer reports from consumers.

### Analysis of fulvestrant AEs risk signals

In this study, a total of 45 new AEs related to fulvestrant were identified through the FAERS database, involving 11 different systems and organs. Among these were risk signals related to respiratory, thoracic, and mediastinal disorders, encompassing interstitial lung disease and acute interstitial pneumonitis. While drug-induced interstitial lung disease with fulvestrant was relatively rare, there are currently no documented reports in the literature^17^. However, our study found that fulvestrant may potentially trigger drug-induced interstitial lung disease (ROR = 4.85, PRR = 4.81) and acute interstitial pneumonitis (ROR = 50.55, PRR = 50.51). Therefore, when patients undergoing fulvestrant treatment experience interstitial lung disease or respiratory symptoms, healthcare providers should consider the association with fulvestrant.

Risk signals associated with general conditions and administration site reactions, including injection site discomfort, injection site edema, and administration site pain, align with the descriptions in the drug’s label. However, in our study, we found a strong signal for injection site necrosis (ROR = 46.78, PRR = 46.58), which was not mentioned in the drug label. Murdock^18^reported a detailed case of injection site necrosis following fulvestrant administration. This case highlighted that patients may experience injection site necrosis when receiving fulvestrant injections and may require antibiotic and corticosteroid treatment if necessary.

The risk signal associated with vascular and lymphatic disorders is the most common adverse reaction of fulvestrant, with platelet count decreased having the highest incidence rate. AEs that were not mentioned in the drug label include pancytopenia、full blood count decreased、bone marrow failure. Hematologic toxicity is a common adverse reaction to fulvestrant, and if any of the above AEs are observed after drug administration, appropriate symptomatic treatment should be given in a timely manner.

A common adverse reaction associated with skin and subcutaneous tissue disorders is alopecia and dry skin. Our study found that skin toxicity is a strong signal (n = 14, ROR = 367.47) that has not been mentioned in the drug label. Our study found that the skin toxicity of fulvestrant mainly includes rash, itching, hair loss, and palmar-plantar erythrodysaesthesia syndrome, and we did not find toxic epidermal necrolysis. However, Morales-Conde^19^ reported a case of fulvestrant-induced toxic epidermal necrolysis. The patient mainly presented with rashes, blisters, and skin peeling. Therefore, when patients receive fulvestrant treatment, if they develop toxic epidermal necrolysis, prompt administration of corticosteroids and intravenous immunoglobulin (IVIG) should be considered^20^.

In addition to the above risk signals, during the clinical use of fulvestrant, special attention should also be paid to jaundice, biliary stenosis, liver necrosis, hypertensive crisis, circulatory failure, hyperglycemia, hypokalemia, and hypercalcemia, which are risk signals not yet recorded in the drug’s label. Enhanced patient monitoring, regular laboratory tests, timely adjustments, and ensuring patient medication safety are crucial.

### Analysis of factors influencing severe AEs of fulvestrant

#### Age

Currently, there are no research reports on age-related AEs associated with fulvestrant. Our study results indicateed that the risk of severe fulvestrant-related AEs in patients aged 60–74 is 0.521 times that of the 0–44 age group. Age served as a protective factor for severe fulvestrant-related AEs. This may be related to the clinical characteristics and therapeutic regimen of pre- and post-menopausal breast cancer patients. Previous studies have shown that compared to postmenopausal breast cancer, premenopausal breast cancer is often more invasive with larger tumors, a higher proportion of lymph node positivity, higher histological grade, and therefore often has a higher risk of recurrence, worse prognosis, and higher mortality rates^21^. Premenopausal breast cancer patients are more likely to receive combined treatment with multiple cancer drugs, such as chemotherapy combined with hormone therapy^22^. Compared to postmenopausal breast cancer patients using hormone therapy, the increase in cancer drugs in premenopausal breast cancer patients makes them more prone to adverse reactions. The increase in adverse reactions in patients aged 75 and older may be related to their declining metabolic and excretory functions, leading to drug accumulation in the body and resulting in higher adverse reactions^23^.

#### CYP3A4 enzyme inhibitors

A meta-analysis has indicated that the likelihood of drug interactions between fulvestrant and CYP3A4 enzyme inhibitors is minimal, and the occurrence rate of adverse reactions is unlikely to increase significantly when these were used in combination^24^. Based on the reported literature and clinical studies, only a few interactions between fulvestrant and CYP3A4 inhibitors such as ketoconazole and midazolam have been mentioned, and there is a lack of large-sample and diverse CYP3A4 enzyme inhibitor-related data studies^25,26^. In our current study, we conducted a statistical analysis of concomitant drug, and the results revealed 134 reported cases of fulvestrant being used in conjunction with CYP3A4 enzyme inhibitors. The primary co-administered medications included verapamil, amiodarone, itraconazole, clarithromycin, and amlodipine. To confirm the correlation between fulvestrant combination with CYP3A4 enzyme inhibitors and AEs, we conducted an analysis of the factors influencing CYP3A4 enzyme inhibitors. The results indicated that CYP3A4 enzyme inhibitors as concomitant medications are not an independent risk factor for severe AEs with fulvestrant [OR = 1.372 (0.886, 2.126), *p* = 0.156], and this finding is consistent with previous reports^24–26^.

#### Protein binding rate

Fulvestrant binds to various targets in the blood, such as very low-density lipoprotein, low-density lipoprotein, high-density lipoprotein, with a plasma protein binding rate as high as 99%. Pharmacokinetically, it is presumed that when two or more high protein-binding drugs are used simultaneously, they competitively bind to each other, leading to an increase in the free component of fulvestrant, thereby significantly increasing AEs. When two drugs with high protein binding rates are co-administered, it is controversial whether the competition for protein binding sites leads to an increase in the concentration of free drugs and whether this interaction has clinical significance^27,28^.

Currently, there is no definite definition regarding the high or low protein binding rates. Drawing from Brigitte’s work on protein binding rates^29^, we categorized the protein binding rates into three groups: extremely high (≥ 98%), high (85%-98%), and moderately low (less than 85%), to explore the correlation between protein binding rates and severe AEs related to fulvestrant. Our study results demonstrated that protein binding rates are an independent risk factor for the occurrence of severe AEs with fulvestrant. Interestingly, we observed a 1.522-fold increased risk of severe adverse events (AEs) when fulvestrant was co-administered with drugs exhibiting extremely high protein binding rates (≥ 98%), compared to the use of fulvestrant alone (OR = 1.522 [1.173, 1.975], *p* = 0.002). When fulvestrant is co-administered with drugs with extremely high protein binding rates (≥ 98%), there may be an increased risk of adverse reactions due to drug interactions. Therefore, healthcare professionals should exercise caution when prescribing fulvestrant in combination with extremely high protein binding drugs. Finally, further clinical research is needed to explore and validate whether fulvestrant interacts with drugs with high protein binding rates and the mechanisms involved.

## Limitations

Our study has limitations: (1) the reported population comes from various industries, and the quality of the reports may vary due to different levels of expertise. (2) The FAERS database does not provide information on the actual population using the drugs, making it difficult to calculate the incidence rate of AEs. (3) The FAERS database relies on voluntary reporting, which may introduce bias. (4) The FAERS database has slow updates, which may result in lagging data. (5) The associated association between fulvestrant and adverse events was based on physician judgment, and we cannot conclude that a causal relationship is real.The occurrence of AEs may be related to underlying conditions being treated, concomitant use of other medications, or reasons not yet understood. (6) We cannot provide strong evidence to demonstrate the potential biological mechanisms between fulvestrant and AEs. (7) Due to the exploratory nature of our study, our findings must be validated through prospective research.

## Conclusion

As of September 30, 2023, we have received a total of 6947 reports on fulvestrant through the FAERS database. We identified 210 valid risk signals, including 45 new AEs. The logistic regression results indicated that the protein binding rate is a risk factor for severe AEs associated with fulvestrant, while age serves as a protective factor. Furthermore, CYP3A4 enzyme did not increase the risk of severe AEs associated with fulvestrant. Due to the exploratory nature of our study, our findings must be validated through prospective research. In the future, more research is needed to help determine the relationship between fulvestrant and protein binding rates, and to fully elucidate any potential biological mechanisms that may exist, thereby enhancing risk management efforts.

### Supplementary Information

Supplementary Information.

## Data Availability

The original contributions proposed in this study are included in the article/supplementary materials. For further inquiries, please contact the corresponding author directly.
