# Supplementary material for: Adverse event signal mining and serious adverse event influencing factor analysis of fulvestrant based on FAERS database
Source: Sci Rep. 2024 May 18;14:11367. doi: 10.1038/s41598-024-62238-1 (PMC11102440; doi:10.1038/s41598-024-62238-1)
Supplement: Supplementary file 1 — Supplementary Information. [file 41598_2024_62238_MOESM1_ESM.docx]

**Supplements:**

**Adverse Event Signal Mining and Serious Adverse Event Influencing Factor Analysis of fulvestrant Based on FAERS Database**

**Guisen Yin^2*^, Guiling Song^3^, Shuyi Xue^4^, Fen Liu^1^**🖂

^1^ Department of Pharmacy, The Affiliated Cancer Hospital of Xiangya School of Medicine, Central South University/Hunan Cancer Hospital, Changsha 410011, Hunan, China

^2^ Department of Pharmacy, Yantai Hospital of Traditional Chinese Medicine, Yantai 264000, Shandong, China

^3^ Department of Chemical medicine, Yantai Center For Food And Drug Control, Yantai 264003, Shandong, China

^4^ Department of Pharmacy, Qingdao Central Hospital, University of Health and Rehabilitation Sciences(Qingdao Central Medical Group), Qingdao 266042, Shandong, China

🖂Corresponding author:Fen Liu,E-mail:[liufen@hnca.org.cn](mailto:panyong@hnca.org.cn.Abstract)

**Table S1.** **Two-by-two contingency table for analyses.**

| Drug | Target adverse events | Non-target adverse events | total |
| --- | --- | --- | --- |
| Fulvestrant | a | b | a+b |
| All other drugs of interest | c | d | c+d |
| Total | a+c | b+d | a+b+c+d |

Equation: a,number of reports containing both the target drug and target adverse drug reaction; b, number of reports containing other adverse drug reaction of the target drug; c, number of reports containing the target adverse drug reaction of other drugs; d, number of reports containing other drugs and other adverse drug reactions.

**Table S2. Summary of major algorithms used for signal detection.**

| Method | Formula | Threshold |
| --- | --- | --- |
| ROR | $ROR=\frac{a}{b}\div\frac{c}{d}$ = $\frac{\mathrm{ad}}{\mathrm{bc}}$  ${95\%CI=e}^{lnROR\pm1.96\sqrt{\frac{1}{a} +\frac{1}{b} + \frac{1}{c} + \frac{1}{d}}}$ | N≥3，95%CI下限＞1 |
| PRR | $PRR=\frac{a}{a+c}\div\frac{b}{b+d}$ = $\frac{a(b+d)}{(a+c)b}$  ${95\%CI=e}^{lnROR\pm1.96\sqrt{\frac{1}{a} +\frac{1}{b} + \frac{1}{c} + \frac{1}{d}}}$  χ^2^= $\frac{(ad-bc)(ad-bc)(a+b+c+d)}{(a+b)(c+d)(b+d)(a+c)}$ | N≥3，PRR＞2，χ^2^≥4 |

Equation: ROR, reporting odds ratio; CI, confidence interval; PRR, proportional reporting ratio; 95%CI, 95% confidence interval; N, the number of reports; χ^2^,chi-squared;

**Table S3.** **Signal strength of reports of fulvestrant at the Preferred Term (PT) level in FAERS database.**

| SOC | Preferred Terms (PTs) | Reports | ROR | PRR (χ2) |
| --- | --- | --- | --- | --- |
| Blood and lymphatic system disorders | neutropenia | 325 | 10.30 (9.2-11.52) | 9.80(2559.28) |
|  | anaemia | 150 | 3.41(3.48-2.96) | 3.42(1255.36) |
|  | thrombocytopenia | 126 | 4.80(4.89-4.10) | 4.81(376.89) |
|  | leukopenia | 114 | 9.84 (8.35-12.11) | 9.89 (898.56) |
|  | white blood cell count decreased | 113 | 4.25(3.53-5.12) | 4.19(271.69) |
|  | neutrophil count decreased | 87 | 9.53(7.71-11.78) | 9.41(642.92) |
|  | platelet count decreased | 56 | 2.17(1.67-2.82) | 2.16(33.75) |
|  | myelosuppression | 36 | 6.79(4.89-9.42) | 6.75(170.22) |
|  | Pancytopenia* | 32 | 2.50(1.76-3.54) | 2.49(27.08) |
|  | red blood cell count decreased | 27 | 4.23(2.90,6.18) | 4.23(63.00) |
|  | blood count abnormal | 18 | 3.57(2.25,5.67) | 3.56(30.59) |
|  | full blood count decreased* | 16 | 9.81(4.89-19.65) | 9.80(54.41) |
|  | bone marrow failure* | 8 | 3.21(2.02,5.10) | 3.21(25.12) |
| Cardiac disorders | pericardial effusion* | 16 | 2.84(1.74-4.65) | 2.84(17.26) |
|  | bundle branch block left* | 4 | 3.78(1.42-10.08) | 3.78(5.61) |
|  | intracardiac thrombus* | 4 | 3.90(1.46-10.40) | 3.90(5.95) |
| Gastrointestinal disorders | ascites* | 59 | 8.51(6.58,11.01) | 8.44(378.20) |
|  | gastritis | 16 | 2.58(1.58,4.21) | 2.57(13.82) |
|  | oral pain | 13 | 2.31(1.34,3.99) | 2.31(8.40) |
|  | frequent bowel movements* | 11 | 2.10(1.16,3.80) | 2.10(5.28) |
|  | aphthous ulcer | 10 | 4.70(2.53,8.75) | 4.70(25.46) |
|  | Eructation* | 10 | 2.33(1.25,4.33) | 2.32(6.27) |
|  | small intestinal obstruction* | 9 | 3.47(1.80,6.68) | 3.47(13.42) |
|  | paraesthesia oral | 8 | 2.27(1.13,4.55) | 2.27(4.49) |
| General disorders and administration site conditions | injection site pain | 205 | 2.65(2.30,3.04) | 2.59(201.19) |
|  | asthenia | 186 | 2.09(1.81,2.42) | 2.06(101.31) |
|  | injection site mass | 52 | 5.25(4.00,6.90) | 5.22(172.75) |
|  | injection site necrosis | 27 | 46.78(31.91,68.59) | 46.58(113.16) |
|  | mucosal inflammation | 25 | 4.30(2.91,6.38) | 4.29(59.77) |
|  | injection site nodule | 22 | 7.21(4.74,10.96) | 7.19(110.67) |
|  | injection site extravasation | 20 | 4.92(3.17,7.62) | 4.91(58.33) |
|  | injection site discomfort | 14 | 5.32(3.15,9.00) | 5.31(44.70) |
|  | injection site induration | 13 | 3.55(2.06,6.11) | 3.55(21.20) |
|  | injection site inflammation | 8 | 6.73(3.36,13.47) | 6.72(33.32) |
|  | injection site scar | 8 | 9.94(4.96,19.93) | 9.93(55.34) |
|  | injection site hypoaesthesia | 7 | 20.87(9.91,43.98) | 20.85(111.94) |
|  | injection site nerve damage | 7 | 125.84(58.53,270.58) | 125.7(699.09) |
|  | injection site ulcer | 7 | 28.85(13.67,60.885) | 28.82(158.75) |
|  | embolia cutis medicamentosa | 3 | 12.66(4.07,39.42) | 12.65(21.45) |
|  | injection site oedema | 3 | 7.06(2.27,21.93) | 7.05(10.08) |
| Infections and infestations | erysipelas* | 10 | 9.53(5.12-17.75) | 9.52(67.62) |
|  | injection site abscess* | 9 | 13.75(7.13-26.50) | 13.73(93.20) |
|  | injection site cellulitis* | 5 | 10.45(4.34-25.17) | 10.44(33.57) |
|  | injection site infection | 5 | 6.00(2.49,14.44) | 5.99(16.06) |
|  | Pustule* | 4 | 10.45（3.91-27.93） | 10.45（25.23） |
| Investigations | aspartate aminotransferase increased | 53 | 4.14(3.16,5.43) | 4.11(121.72) |
|  | alanine aminotransferase increased | 47 | 3.12(2.34,4.16) | 3.10(64.87) |
|  | hepatic enzyme increased | 43 | 2.65(1.96,3.58) | 2.64(42.11) |
|  | blood creatinine increased | 42 | 2.57(1.90,3.48) | 2.56(38.46) |
|  | transaminases increased | 37 | 6.51(4.71,8.90) | 6.47(165.39) |
|  | gamma-glutamyltransferase increased | 34 | 6.70(4.78,9.40) | 6.67(157.90) |
|  | blood bilirubin increased | 33 | 4.80(3.41,6.76) | 4.78(94.64) |
|  | electrocardiogram QT prolonged | 31 | 2.97(2.09,4.23) | 2.96(38.27) |
|  | blood alkaline phosphatase increased | 22 | 3.58(2.36,5.45) | 3.57(38.17) |
|  | oestradiol increased | 14 | 5.13(3.03,8.67) | 5.12(42.30) |
|  | ejection fraction decreased* | 10 | 2.38（1.28-4.42） | 2.38（6.64） |
|  | glomerular filtration rate decreased* | 9 | 3.48（1.81-6.70） | 3.48（13.47） |
|  | blood calcium increased* | 8 | 3.73（1.86-7.46） | 3.72（13.30） |
|  | hormone level abnormal | 5 | 4.13(1.72,9.95) | 4.13(8.92) |
|  | blood oestrogen increased | 4 | 28.59(10.65,76.76) | 28.57(79.42) |
|  | alanine aminotransferase abnormal | 3 | 7.13(2.29,22.15) | 7.12(10.22) |
|  | aspartate aminotransferase abnormal | 3 | 10.18(3.27,31.66) | 10.17(16.40) |
|  | creatinine renal clearance increased | 3 | 14.36(4.61,44.74) | 14.36(24.92) |
|  | fibrin d dimer increased | 3 | 4.26(1.37,13.23) | 4.26(4.57) |
| Metabolism and nutrition disorders | decreased appetite | 146 | 2.62(2.22,3.09) | 2.58(140.82) |
|  | hyperglycaemia* | 67 | 7.45(5.85-9.48) | 7.38(362.00) |
|  | hypokalaemia* | 22 | 2.17(1.43-3.30) | 2.17(12.67) |
|  | iron deficiency* | 7 | 8.26(3.93-17.36) | 8.25(27.48) |
|  | polydipsia* | 6 | 5.54(2.48-12.34) | 5.53(17.92) |
| Musculoskeletal and connective tissue disorders | back pain | 115 | 2.06(1.71,2.47) | 2.04(60.01) |
|  | bone pain | 80 | 5.42(4.34,6.76) | 5.36(279.09) |
|  | musculoskeletal pain | 35 | 2.70(1.93,3.76) | 2.69(35.36) |
|  | osteonecrosis of jaw | 20 | 2.86(1.85,4.44) | 2.86(22.31) |
|  | musculoskeletal chest pain | 17 | 4.74(2.95,7.64) | 4.73(46.31) |
|  | jaw disorder | 8 | 4.50(2.25,9.01) | 4.49(18.33) |
|  | groin pain | 7 | 3.48(1.66,7.32) | 3.48(10.01) |
|  | spinal pain | 6 | 2.54(1.14,5.65) | 2.54(4.15) |
| Nervous system disorders | hypoaesthesia | 80 | 2.08(1.67,2.59) | 2.06(43.00) |
|  | neuropathy peripheral | 63 | 2.73(2.13,3.51) | 2.72(66.76) |
|  | sciatica | 24 | 8.12(5.43,12.14) | 8.09(141.66) |
|  | neuralgia | 17 | 3.00(1.87,4.84) | 3.00(20.65) |
|  | polyneuropathy | 17 | 6.63(4.12,10.68) | 6.61(75.26) |
|  | taste disorder | 17 | 4.59(2.85,7.39) | 4.58(43.93) |
|  | brain oedema | 12 | 3.72(2.11,6.56) | 3.72(21.14) |
|  | parosmia | 8 | 4.40(2.20,6.56) | 4.40(17.69) |
|  | peroneal nerve palsy | 6 | 4.11(1.84,9.15) | 4.10(11.13) |
|  | dysaesthesia | 4 | 6.24(2.34,16.65) | 6.23(12.69) |
|  | vocal cord paralysis | 3 | 6.59(2.12,20.47) | 6.59(9.14) |
| Psychiatric disorders | eating disorder | 15 | 2.72(1.64,4.51) | 2.71(14.56) |
| Renal and urinary disorders | urine odour abnormal | 21 | 13.99(9.10,21.51) | 13.95(237.91) |
|  | hydronephrosis | 10 | 5.61(3.02,10.45) | 5.61(33.28) |
|  | bladder discomfort | 3 | 10.11(3.25,31.44) | 10.10(16.25) |
|  | calcinosis | 3 | 5.36(1.73,16.65) | 5.36(6.70) |
| Respiratory, thoracic and mediastinal disorders | cough | 183 | 2.88(2.48,3.33) | 2.82(215.22) |
|  | pulmonary embolism | 82 | 3.01(2.42,3.74) | 2.98(106.20) |
|  | interstitial lung disease* | 54 | 4.85(3.71-6.34) | 4.82(159.40) |
|  | Epistaxis* | 47 | 2.51(1.88-3.34) | 2.50(40.78) |
|  | dyspnoea exertional | 30 | 3.88(2.71,5.56) | 3.87(60.84) |
|  | throat irritation | 25 | 2.32(1.57,3.44) | 2.32(17.45) |
|  | throat tightness | 22 | 3.19(2.10,4.85) | 3.18(30.79) |
|  | pneumonitis | 19 | 3.21(2.05,5.05) | 3.21(26.68) |
|  | Bronchospasm* | 14 | 4.23(2.50-7.15) | 4.22(31.24) |
|  | pulmonary mass | 14 | 4.89(2.89,8.27) | 4.88(39.36) |
|  | acute respiratory failure* | 9 | 2.20(1.14-4.23) | 2.20(4.72) |
|  | laryngospasm* | 9 | 13.69(7.10-26.39) | 13.67(92.76) |
|  | upper-airway cough syndrome | 8 | 3.40(2.00,8.00) | 3.99(15.06) |
|  | oropharyngeal discomfort | 8 | 3.57(1.70,7.51) | 3.57(10.50) |
|  | acute interstitial pneumonitis* | 7 | 50.55(22.46-113.78) | 50.51(237.37) |
|  | pleural thickening | 6 | 52.43(23.29,118.04) | 52.38(246.31) |
|  | laryngeal oedema* | 5 | 3.25(1.35-7.82) | 3.25(5.69) |
|  | painful respiration* | 3 | 5.14(1.65-15.95) | 5.13(6.26) |
| Skin and subcutaneous tissue disorders | alopecia | 119 | 2.33(1.94,2.79) | 2.30(87.06) |
|  | dry skin | 34 | 2.03(1.45,2.84) | 2.02(16.56) |
|  | palmar-plantar erythrodysaesthesia syndrome* | 16 | 2.49(1.52-4.07) | 2.49(12.75) |
|  | skin toxicity | 14 | 10.96(6.48,18.55) | 10.94(116.04) |
|  | skin odour abnormal* | 10 | 9.64(5.18-17.95) | 9.63(68.56) |
|  | skin reaction | 9 | 2.54(1.32,4.88) | 2.54(6.91) |
|  | decubitus ulcer | 7 | 3.75(1.78,7.87) | 3.74(11.44) |
|  | skin hypopigmentation* | 5 | 16.24(6.73-39.18) | 16.23(56.53) |
|  | rash vesicular* | 4 | 3.68(1.38-9.83) | 3.68(5.35) |
|  | skin indentation* | 4 | 39.51(14.67-106.39) | 39.49(111.68) |
|  | Trichorrhexis* | 3 | 4.24(1.37-13.16) | 4.24(4.52) |
| Vascular disorders | hot flush | 79 | 4.46(3.57,5.57) | 4.41(205.25) |
|  | lymphoedema* | 18 | 11.71(7.36-18.63) | 11.68(164.27) |
|  | hypertensive crisis* | 16 | 5.53(3.38-9.03) | 5.52(54.58) |
|  | circulatory collapse* | 12 | 2.63(1.49-4.64) | 2.63(10.53) |
|  | embolism | 7 | 2.89(1.38,6.07) | 2.89(6.84) |
|  | phlebitis | 5 | 4.07(1.69,9.80) | 4.07(8.69) |
|  | vena cava thrombosis | 4 | 9.93(3.72,26.53) | 9.92(23.67) |
|  | jugular vein thrombosis | 3 | 7.48(2.41,23.24) | 7.47(10.92) |
| Hepatobiliary disorders | hepatotoxicity | 30 | 4.83(3.37,6.92) | 4.81(86.69) |
|  | Jaundice* | 26 | 3.59(2.44-5.28) | 3.58(45.68) |
|  | hepatic function abnormal | 19 | 2.12(1.25,3.33) | 2.12(10.13) |
|  | hepatic lesion | 19 | 18.82(11.97,29.60) | 18.77(299.12) |
|  | hepatomegaly* | 13 | 5.16(2.99-8.90) | 5.15(39.32) |
|  | liver function test increased | 13 | 2.65(1.54,4.57) | 2.65(11.72) |
|  | acute hepatic failure | 10 | 2.70(1.45,5.02) | 2.69(9.02) |
|  | hyperbilirubinaemia | 9 | 3.85(2.00,7.40) | 3.84(16.16) |
|  | hypercalcaemia | 9 | 3.26(1.69,6.27) | 3.25(11.87) |
|  | portal vein thrombosis | 6 | 7.17(3.21,15.98) | 7.16(25.84) |
|  | bile duct stenosis* | 5 | 19.62(8.12-47.36) | 19.60(69.90) |
|  | hepatic cyst* | 4 | 5.29(1.98-14.11) | 5.29(9.91) |
|  | hepatic necrosis* | 4 | 3.83(1.44-10.22) | 3.83(5.76) |
|  | hypertransaminasaemia | 4 | 3.57(1.34,9.54) | 3.57(5.05) |
|  | hepatitis fulminant | 3 | 4.07(1.31,12.64) | 4.07(4.21) |
| immune system disorders | decreased immune responsiveness* | 9 | 4.31(2.24,8.30) | 4.31(19.62) |
|  | anaphylactoid reaction | 6 | 5.57(2.50,12.43) | 5.57(18.11) |

^*^Emerging findings of fulvestrant associated AEs from FAERS database.
